# Causal Relationship Between Gut Microbiota and Benign Prostatic Hyperplasia: A Two‐Sample Mendelian Randomization Analyses, 16S rRNA Sequencing and Clinical Retrospective Study

**DOI:** 10.1002/fsn3.71261

**Published:** 2025-11-21

**Authors:** Ting‐Ting Lin, Bo‐Han Lin, Cheng‐Long Zeng, Zhong‐Hua Zhu, Jun‐Ming Zhu, Yang Chen, Shao‐Hao Chen, Qing‐Shui Zheng, Xue‐Yi Xue, Yong Wei, Ning Xu, Ye‐Hui Chen, Yi‐Cheng Xu

**Affiliations:** ^1^ Department of Urology, Urology Research Institute, the First Affiliated Hospital Fujian Medical University Fuzhou China; ^2^ Department of Urology, National Regional Medical Center, Binhai Campus of the First Affiliated Hospital Fujian Medical University Fuzhou China; ^3^ Fujian Key Laboratory of Precision Medicine for Cancer, the First Affiliated Hospital Fujian Medical University Fuzhou China

**Keywords:** benign prostatic hyperplasia, causal relationship, *Escherichia*–*Shigella*, gut microbiota, Mendelian randomization

## Abstract

Recent studies suggest potential associations between gut microbiota (GM) and benign prostatic hyperplasia (BPH); however, the causal relationship remains uncertain. This study aims to explore the potential causal links between GM and BPH through Mendelian randomization (MR) analysis. A two‐sample MR analysis was conducted using data from genome‐wide association studies in accordance with the MR‐STROBE statement. The inverse variance–weighted (IVW) method was utilized as the primary estimator. Robustness was evaluated by heterogeneity testing, horizontal pleiotropy assessment, and leave‐one‐out analyses. Fecal samples from BPH patients and healthy controls underwent 16S rRNA sequencing to evaluate their association with lower urinary tract symptom (LUTS) severity. IVW analyses associated *Phascolarctobacterium* (OR = 1.286; 95% CI, 1.023–1.618; *p* = 0.031), *Faecalibacterium* (OR = 1.134; 95% CI, 1.008–1.275; *p* = 0.037), and *Escherichia*–*Shigella* (OR = 1.348; 95% CI, 1.121–1.621; *p* = 0.002) with higher BPH risk, while *Lactobacillus* (OR = 0.572; 95% CI, 0.412–0.793; *p* < 0.001) and *Burkholderia* (OR = 0.718; 95% CI, 0.565–0.912; *p* = 0.007) were protective. WM and MR‐Egger confirmed only *Escherichia*–*Shigella* as consistently associated. Reverse MR found no causal effect of BPH on these taxa, with no heterogeneity or pleiotropy detected. 16S rRNA sequencing showed greater *Escherichia*–*Shigella* abundance in BPH patients than controls. Multivariate analysis identified *Escherichia*–*Shigella* relative abundance as an independent predictor of severe LUTS (OR = 1.10; 95% CI, 1.01–1.21; *p* = 0.046). ROC analysis demonstrated its predictive value for LUTS severity (AUC = 0.777; 95% CI, 0.649–0.876; *p* < 0.001). Our study supports a potential causal role of GM in BPH, with *Escherichia*–*Shigella* emerging as a key predictor of LUTS severity. These findings may provide insights for future therapeutic interventions targeting microbial dynamics in BPH treatment.

## Graphical abstract

Our study supports a potential causal role of gut microbiota in benign prostatic hyperplasia, with *Escherichia*–*Shigella* emerging as a key contributor and predictor of lower urinary tract symptom severity. These findings may provide insights for future therapeutic interventions targeting microbial dynamics in the treatment of benign prostatic hyperplasia.

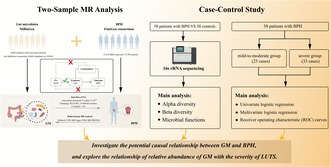

## 1 Introduction

Benign prostatic hyperplasia (BPH) is the most prevalent benign neoplasm in men of middle age and older, characterized by symptoms such as frequent urination, urgency, and increased nighttime urination (Devlin et al. 2021). The condition impacts 8% of men in their 40s, increasing to 90% in their 80s (Langan 2019). BPH frequently leads to lower urinary tract symptoms (LUTS), which notably affect patients' quality of life. Although its etiology remains unclear, recognized risk factors include testosterone levels and aging (B. Li, Chen, et al. 2024). In addition, androgen and its receptor actions, imbalance between cell proliferation and apoptosis, and growth factor involvement are also associated with the pathogenesis of BPH (Chung et al. 2024).

In recent years, numerous studies have indicated a possible link between the gut microbiota (GM) and BPH (An et al. 2023; Russo et al. 2023). GM is essential for human health, influencing immune reactions, metabolic functions, development, and various physiological activities. It is also associated with the progression of multiple diseases, including periodontal disease, cancer, diabetes, liver disease, inflammatory bowel disease, and obesity‐related disorders (Ditto et al. 2021; Lopetuso et al. 2023). However, various studies have linked differences between healthy and bioimbalanced GM to BPH. Gu et al. (Tsai et al. 2022) reported that high‐fat diet‐induced GM alterations promoted BPH development. Takezawa et al. (2021) observed significant shifts in GM composition in BPH patients, with *Firmicutes* and *Bacteroidetes* markedly enriched. Yang et al. (2024) suggested that host‐mediated modulation of specific GM and engagement of multiple metabolic pathways may underlie the therapeutic effects of Xiaojin pill in BPH. Collectively, these findings indicate that GM alterations may influence the pathogenesis, diagnosis, and early prevention of BPH, while causal relationships remain undetermined.

Although RCTs are regarded as the gold standard for establishing causality, no studies have investigated the relationship between GM and BPH. In Mendelian randomization (MR), genetic variants are used as instrumental variables (IVs) to assess causality between exposures and outcomes. Since alleles are fixed at conception and randomly assorted according to Mendelian principles, MR is less vulnerable to environmental confounding and reverse causation (Ouyang et al. 2024). Consequently, MR is widely applied as an alternative to RCTs in causal inference research.

In our research, we utilized a two‐sample MR approach to explore the causal links between specific GM and BPH risk. Subsequently, we employed 16S rRNA gene sequencing to evaluate the relative abundance of GM confirmed by MR analysis in BPH patients. Finally, we explored its relationship with the severity of LUTS, aiming to reveal new aspects of BPH pathogenesis and aid in formulating new preventive and therapeutic strategies.

## 2 Material and Methods

### 2.1 Ethical Statement

This study was approved by the Ethics Committee of the First Affiliated Hospital of Fujian Medical University. A written informed consent was signed by all BPH patients and healthy volunteers for experiments using the clinical samples.

### 2.2 Research Design

**FIGURE 1:**
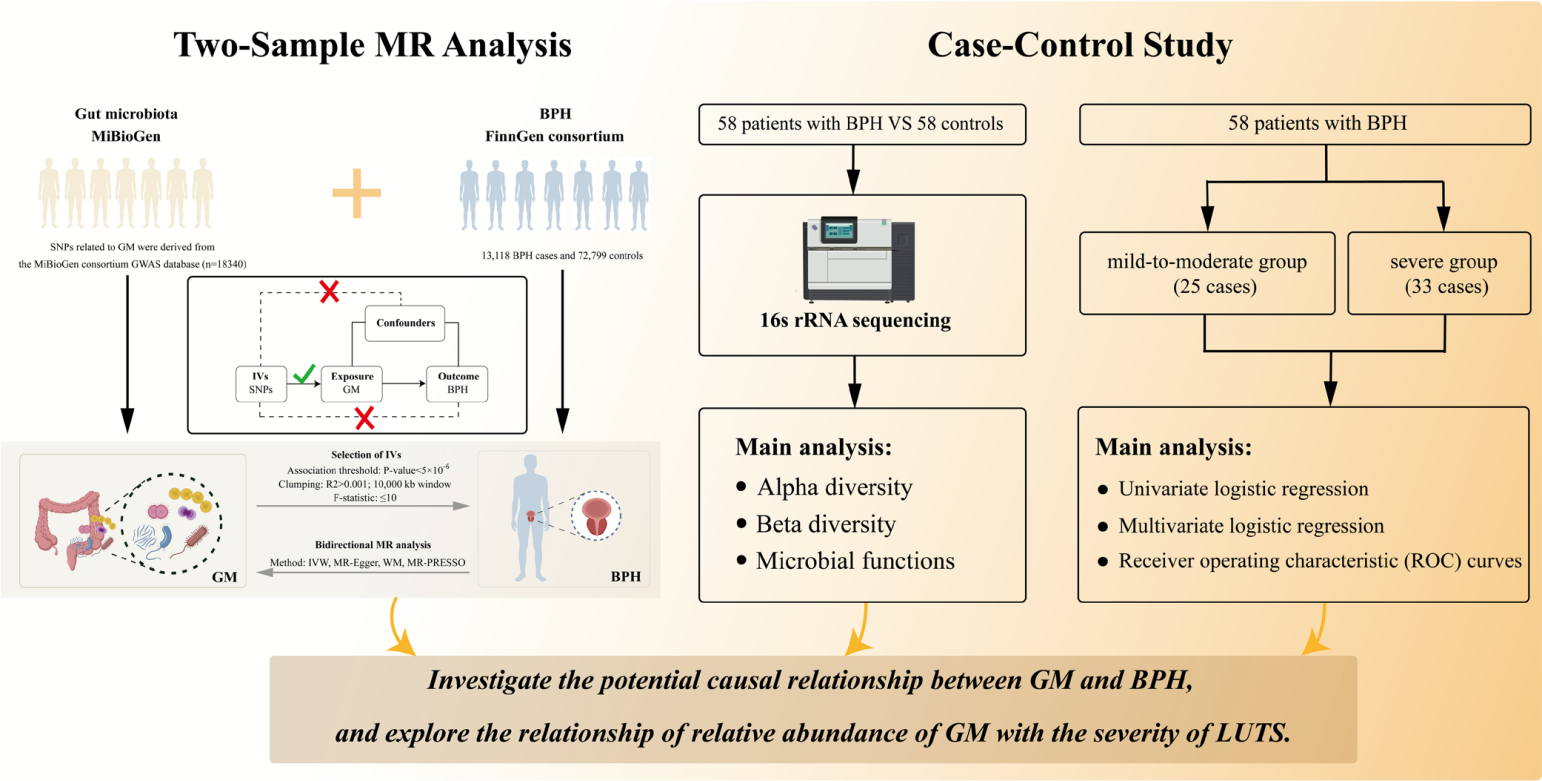
Flow diagram depicting the design and analysis procedure of the study. BPH, benign prostatic hyperplasia; GM, gut microbiota; IV, instrumental variables; IVW, inverse‐variance weighted; LUTS, lower urinary tract symptoms; MR, Mendelian randomization; SNP, single nucleotide polymorphism; WM, weighted median.

As shown in Figure 1, our study outlines a thorough framework to elucidate the genetic causal relationship between GM and BPH. Our study considered GM and BPH as exposure factors and outcomes, respectively. Upon verification of the independence of summary data from genome‐wide association studies (GWAS) for both exposure and outcome variables, we first conducted a two‐sample MR analysis to assess the causal relationships between GM and BPH, as shown in Figure 1. Summary statistics for GM and BPH were downloaded from GWAS. After selecting the appropriate IVs, MR analysis was executed, and sensitivity analyses were performed to assess the consistency of the results. MR analysis is based on three essential assumptions: (1) IVs must be strongly associated with the exposure; (2) IVs should be independent of confounders; and (3) IVs should influence the outcome solely through their effect on the exposure (Verduijn et al. 2010). The MR analysis was performed in accordance with the MR‐STROBE statement. The STROBE‐MR checklist was provided in Table S1 (Skrivankova et al. 2021).

Subsequently, fecal samples from 58 BPH patients and 58 healthy male controls, admitted to the First Affiliated Hospital of Fujian Medical University between September 2024 and May 2025, were collected for 16S rRNA sequencing to validate the MR findings. Moreover, we recorded patients' clinical data on the day of diagnosis, including age, body mass index (BMI), prostate volume (PV), total prostate‐specific antigen (tPSA) level, hypertension, diabetes, C‐reactive protein (CRP), post‐voided residual urine, neutrophil count, lymphocyte count, monocyte count, platelet count, and the systemic inflammatory response biomarkers. The systemic inflammatory response biomarkers were defined as follows: the neutrophil‐lymphocyte ratio (NLR) is the ratio of neutrophil count to lymphocyte count (Valero et al. 2021); the lymphocyte‐monocyte ratio (LMR) is the ratio of lymphocyte count to monocyte count (Valero et al. 2021); the platelet‐lymphocyte ratio (PLR) is the ratio of platelet count to lymphocyte count (Qi et al. 2021); and the systemic inflammation response index (SIRI) is the product of neutrophil count and monocyte count (Zhao et al. 2022). As per the Ellipse formula, the prostate volume in milliliters is calculated as 0.52 multiplied by the height in centimeters, the length in centimeters, and the width in centimeters (Rodriguez Jr. et al. 2008). To shed light on the causal relationship between GM abundance and BPH, we assessed the relative abundance of GM confirmed by MR analysis in BPH patients, and explored its relationship with the severity of LUTS. All participants provided written informed consent for separate studies approved by the ethics committees of the Institutional Review Boards.

### 2.3 Data Source

The summary‐level GWAS data for BPH were sourced from the FinnGen consortium (ID: finn‐b‐N14_PROSTHYPERPLA) (ICD code: ICD10: N40 Hyperplasia of prostate) (Kurki et al. 2023). The dataset includes 85,917 participants of European descent, comprising 13,118 BPH cases and 72,799 controls, and encompasses 16,378,414 single nucleotide polymorphisms (SNPs) (https://gwas.mrcieu.ac.uk/datasets/finn‐b‐N14_PROSTHYPERPLA/).

The summary statistics for SNPs related to GM were derived from the MiBioGen consortium GWAS database (https://mibiogen.gcc.rug.nl/) (Kurilshikov et al. 2021). This multi‐ethnic cohort includes 18,340 participants of primarily European descent from 24 cohorts across 11 countries, with 16S rRNA gene sequencing and genotyping data. Locus‐based analyses identified GM features, including microbial composition, taxonomic groups, and host genetic variations, while adjusting for sex, age, technical covariates, and genetic principal components. The dataset covered 211 gut microbiota taxa, but 15 unclassified taxa left 196 included taxa.

### 2.4 Instrumental Variable Selection

To assess the causal effect of genetic variants on BPH, we first selected SNPs significantly associated with the exposure (*p* < 5 × 10^−6^) as IVs. Subsequently, we removed SNPs in linkage disequilibrium (LD) (*r* ^2^ > 0.001 and kb = 10,000) to avoid LD interference. The outcome dataset was used to extract SNPs linked to the exposure, excluding any directly related to the outcome. Harmonization of the merged dataset was performed to remove SNPs that were palindromic and incompatible. F‐statistics were then calculated for each SNP to assess instrument strength, and variants with *F* ≤ 10 were excluded to decrease the likelihood of weak‐instrument bias.

Three weighting approaches were applied to examine the association between exposures and outcomes: the inverse‐variance weighted (IVW) method, MR‐Egger regression, and the weighted median (WM) method. The IVW method, assuming all IVs are valid, is the primary statistical approach and synthesizes Wald ratio estimates from each SNP to obtain the overall effect size. The other two methods serve as supplements to IVW; when results differ, IVW takes precedence. The weighted median technique prioritizes valid instrumental variables, delivering trustworthy causal estimates despite the presence of some invalid ones. Egger‐intercept and MR‐PRESSO were applied to examine outliers and horizontal pleiotropy, with *p* > 0.05 in MR‐Egger indicating no detectable pleiotropy. A robust causal association is considered when all three methods yield consistent directional results. The odds ratio (OR) is used to assess the causal relationship strength. It represents the relative probability of the outcome variable occurring in the presence of the exposure factor. An OR > 1 indicates that the exposure factor increases the risk of the outcome variable, suggesting a positive causal relationship, while an OR < 1 indicates a protective or inverse causal relationship. It helps determine the direction of the causal relationship between GM and BPH.

**FIGURE 2:**
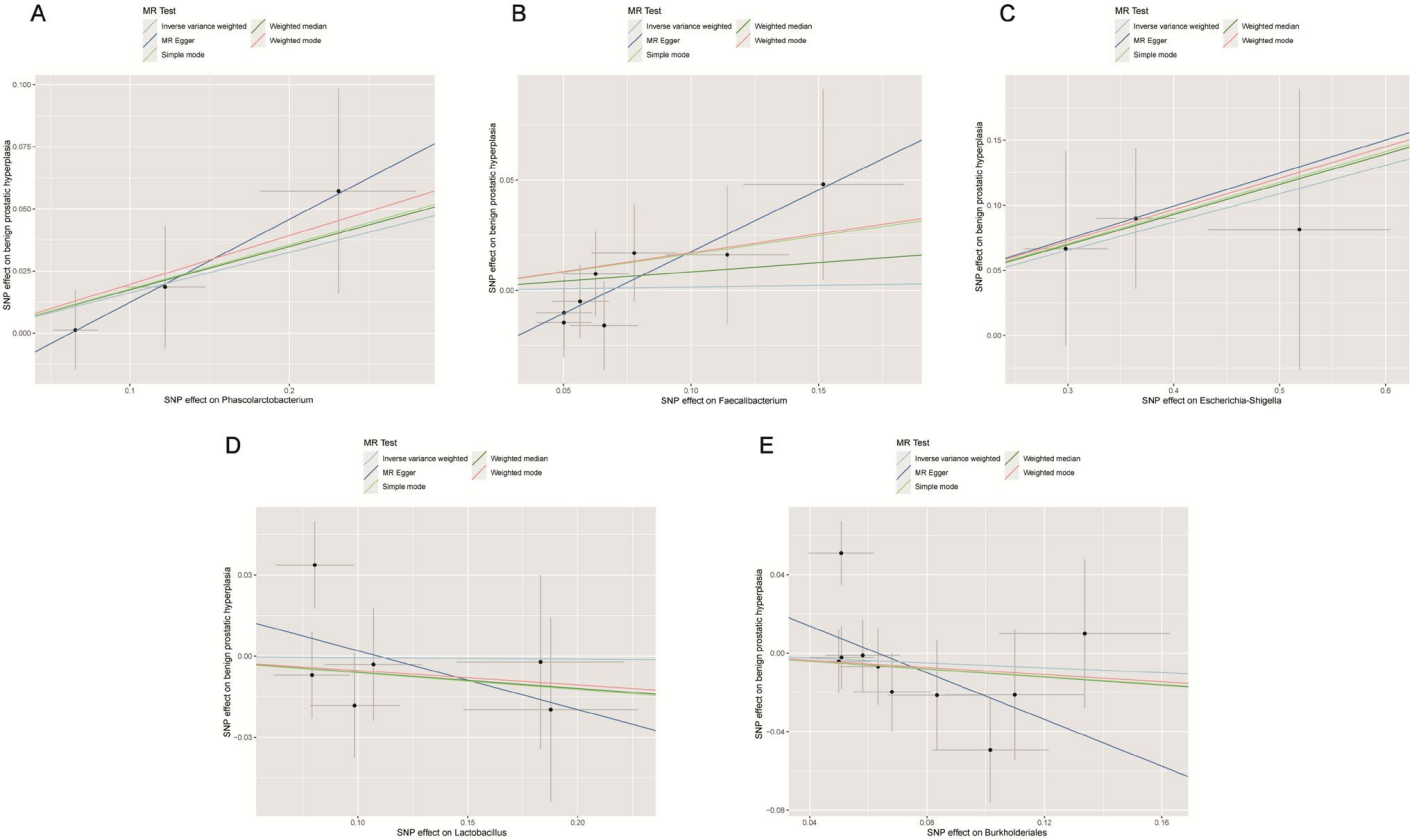
Scatter plot of MR analysis between gut microbiota and BPH. (A) *Phascolarctobacterium*; (B) *Faecalibacterium*; (C) *Escherichia*–*Shigella*; (D) *Lactobacillus*; (E) *Burkholderia*. BPH, benign prostatic hyperplasia; MR, mendelian randomization; SNP, single nucleotide polymorphism.

To further clarify the criteria for identifying significant findings, we defined a causal relationship as statistically significant when the IVW method produced a *p* value < 0.05 and the direction of the effect (β) was consistent across at least one complementary approach (MR‐Egger regression or WM). The IVW method, as the primary estimator, provides the most efficient and unbiased estimates when all IVs are valid. To enhance the reliability of the results, MR‐Egger and WM analyses were conducted as supplementary sensitivity approaches, as they are more tolerant of potential invalid instruments or horizontal pleiotropy. When the direction of β values among different methods was inconsistent, the results were interpreted cautiously. In such cases, the IVW estimate was prioritized due to its higher efficiency under the assumption of no horizontal pleiotropy. However, inconsistent effect directions were viewed as potential indications of bias or pleiotropy, and such associations were not considered robust unless supported by convergent evidence from MR‐Egger intercept, MR‐PRESSO global tests, and heterogeneity analyses. These combined criteria ensured that only stable and biologically plausible causal signals were retained for interpretation.

### 2.5 Reverse MR Analysis

A reverse MR analysis was conducted to further examine the causal relationship between GM and BPH, considering BPH as the exposure factor and GM as the outcome variable, to evaluate the reverse causal effect and reduce confounding bias.

### 2.6 Data Collection, Selection, and Analysis From Network Databases

Gut metagenomic data from BPH patients and healthy controls were downloaded from the NCBI Sequence Read Archive (SRA, https://www.ncbi.nlm.nih.gov/sra) and the European Nucleotide Archive (ENA, https://www.ebi.ac.uk/ena). After standard normalization and filtering, the data were converted into TXT format and imported into Cytoscape for visualization of species co‐occurrence networks. The filtered data were then uploaded to the Peryton database (https://dianalab.e‐ce.uth.gr/peryton/), where a hierarchical diagram was generated to display the correlation between microbiota and disease, with a focus on genus‐level associations. Using the data in TXT format, comparisons were made between disease and healthy populations, highlighting the target microbiota identified in the MR analysis, and assessing the abundance differences of specific microbiota across groups.

### 2.7 Storage of Fecal Samples and Analysis Through 16S rRNA Sequencing

To verify the MR analysis findings, fecal samples were additionally gathered from 58 BPH patients and 58 healthy male controls at our hospital. Middle fecal samples were collected from each participant to minimize variability, and they were immediately frozen at −80°C to ensure their stability and integrity.

Microbial DNA extraction was performed using the HiPure Soil DNA Kit (or HiPure Stool DNA Kit) (Magen, Guangzhou, China), following the manufacturer's protocol. Primers targeting the conserved regions of the 16S rRNA gene were designed, incorporating sample‐specific barcodes for multiplexing. The amplification of the 16S rRNA gene was achieved through PCR, followed by purification and recycling of the PCR products using AMPure XP Beads (Beckman, CA, USA). The purified products were measured with the Quant‐iT PicoGreen dsDNA Assay Kit by Thermo Fisher Scientific (Waltham, MA, USA), and analyzed using a BioTek FLx800 microplate reader (Winooski, VT, USA).

Amplified DNA was sequenced using the MiSeq platform (Illumina, San Diego, CA, USA). The sequencing reads were processed using DADA2 (v1.14.1) for quality filtering, denoising, and splicing, followed by the removal of chimeric sequences. Taxonomic classification of amplicon sequence variants (ASVs) was carried out using the RDP classifier in combination with the SILVA reference database (v138.2). Differences in bacterial taxa from class to genus levels were assessed using linear discriminant analysis (LDA) with effect size estimation (LEfSe), applying thresholds of *p* < 0.05 and an LDA score ≥ 2.

### 2.8 Sensitivity Analysis

To assess the robustness of the results, we performed sensitivity analyses, including tests for heterogeneity, horizontal pleiotropy, and leave‐one‐out analysis. Heterogeneity for each SNP was evaluated using Cochran's Q test (Bowden and Holmes 2019); when *p* < 0.05, a random‐effects model for IVW was implemented (Greco et al. 2015). Horizontal pleiotropy was evaluated using the MR‐PRESSO method, and bias‐inducing SNPs were excluded before re‐estimating causal effects (Verbanck et al. 2018). Leave‐one‐out tests were used to determine how individual SNPs affected the IVW estimate. All analyses were conducted using the “TwoSampleMR” and “MRPRESSO” packages in R (v4.4.1).

### 2.9 LUTS Assessment

We used the International Prostate Symptom Score (IPSS) (Barry et al. 2017) to evaluate the severity of lower urinary tract symptoms (LUTS). This validated seven‐item questionnaire is designed to assess symptoms such as incomplete emptying, frequency, intermittency, urgency, weak stream, straining, and nocturia. Each item is scored from 0 to 5 based on symptom frequency, resulting in a total score range of 0–35, with higher scores indicating a greater symptom burden. In our study, patients were categorized as mild‐to‐moderate group (score 0–19) or the severe group (score ≥ 20).

### 2.10 Statistical Analysis

The comparison of baseline quantitative variables was conducted using the Student's t‐test or the Mann–Whitney *U* test. Normally distributed data are presented as mean ± standard deviation (SD), non‐normally distributed data as median (interquartile range, IQR), and categorical variables as counts (percentages). Differences in categorical data between groups were assessed using the chi‐squared test or Fisher's exact test. Univariate and multivariate logistic regression analyses were performed to identify risk factors associated with severe LUTS. Receiver operating characteristic (ROC) curves, developed by MedCalc version 15.2 (Ostend, Belgium), were utilized to evaluate the predictive capability of GM relative abundance for LUTS severity in BPH patients. The area under the ROC curve (AUC) and 95% confidence intervals (CIs) were calculated. All clinical data were analyzed in RStudio version 4.4.1, with *p* < 0.05 considered statistically significant.

## 3 Results

### 3.1 IV Selection

We evaluated the IVs for 196 bacteria, including 9 phyla, 16 classes, 20 orders, 32 families, and 119 genera. Based on the criteria mentioned, we selected IVs significantly associated with genome‐wide association studies (*p* < 5 × 10^−6^) and independent (*r* ^2^ > 0.001 and kb = 10,000), yielding a total of 1419 IVs.

### 3.2 Two‐Sample MR Analysis

A two‐sample MR analysis of 196 GM taxa identified five with a potential causal relationship to BPH risk, and all the average F values for each microbial community were > 10 (Table 1). IVW analysis identified *Phascolarctobacterium* [odds ratio (OR) = 1.286, 95% CI: 1.023–1.618, *p* = 0.031] (Figure 2A), *Faecalibacterium* (OR = 1.134, 95% CI: 1.008–1.275, *p* = 0.037) (Figure 2B), and *Escherichia*–*Shigella* (OR = 1.348, 95% CI: 1.121–1.621, *p* = 0.002) (Figure 2C) as positively associated with the risk of BPH. In contrast, *Lactobacillus* (OR = 0.572, 95% CI: 0.412–0.793, *p* < 0.001) (Figure 2D) and *Burkholderia* (OR = 0.718, 95% CI: 0.565–0.912, *p* = 0.007) (Figure 2E) exhibited a protective effect on BPH. However, the results were stable solely for *Escherichia*–*Shigella* when employing the weighted median and MR‐Egger methods, underscoring its stable positive associations with BPH.

**TABLE 1:** MR analysis of BPH by five gut microbiota.

Gut microbiota	Method	nSNP	Mean *F* value	Minimum *F* value	OR (95% CI)	*p*
*Phascolarctobacterium*	IVW	3	32.4	14.8	1.286 (1.023–1.618)	0.031
	MR‐Egger	3			1.081 (0.941–1.244)	0.152
	WM	3			1.181 (1.056–1.324)	0.051
*Faecalibacterium*	IVW	8	28.7	12.6	1.134 (1.008–1.275)	0.037
	MR‐Egger	8			1.060 (0.955–1.182)	0.222
	WM	8			1.111 (1.022–1.213)	0.072
*Escherichia*–*Shigella*	IVW	3	35.2	15.4	1.348 (1.121–1.621)	0.002
	MR‐Egger	3			1.251 (1.101–1.425)	0.045
	WM	3			1.296 (1.152–1.454)	0.033
*Lactobacillus*	IVW	6	30.8	11.3	0.572 (0.412–0.793)	< 0.001
	MR‐Egger	6			0.623 (0.481–0.794)	0.082
	WM	6			0.591 (0.476–0.734)	0.025
*Burkholderiales*	IVW	10	27.9	13.5	0.718 (0.565–0.912)	0.007
	MR‐Egger	10			0.752 (0.602–0.953)	0.112
	WM	10			0.735 (0.605–0.884)	0.050

Abbreviations: BPH, benign prostatic hyperplasia; CI, confidence interval; IVW, inverse‐variance weighted; MR, mendelian randomization; OR, odds ratio; SNP, single nucleotide polymorphism; WM, weighted median.

The reverse MR analysis yielded no definitive evidence for a causal association between BPH and the five GM taxa (Table S2). MR‐Egger and WM results were consistent with IVW, supporting the absence of significant causal effects.

### 3.3 Sensitivity Analysis

Cochran's Q test was performed on the five GM taxa potentially causally associated with BPH, and neither the IVW nor Egger‐intercept tests showed significant heterogeneity (Table S3). The Egger‐intercept test (representing the intercept term from MR‐Egger regression and used to assess directional horizontal pleiotropy) indicated no evidence of horizontal pleiotropy between GM and BPH (Table S3). Leave‐one‐out sensitivity analysis revealed no SNPs with a substantial effect on the effect size, indicating the stability of the results (Figure S1). Additionally, the MR‐PRESSO approach was employed to further evaluate whether there were any outliers or horizontal pleiotropy among the IVs. The results showed that MR‐PRESSO did not detect any significant outlier IVs, and none of the models indicated significant horizontal pleiotropy or bias in the estimates. These findings suggest that the selected IVs are appropriate, and the MR estimates are both stable and reliable. The global test results for each microbiota group, as assessed by MR‐PRESSO, yielded *p*‐values > 0.05, with the detailed results provided in Table S3.

### 3.4 Validation of MR

**FIGURE 3:**
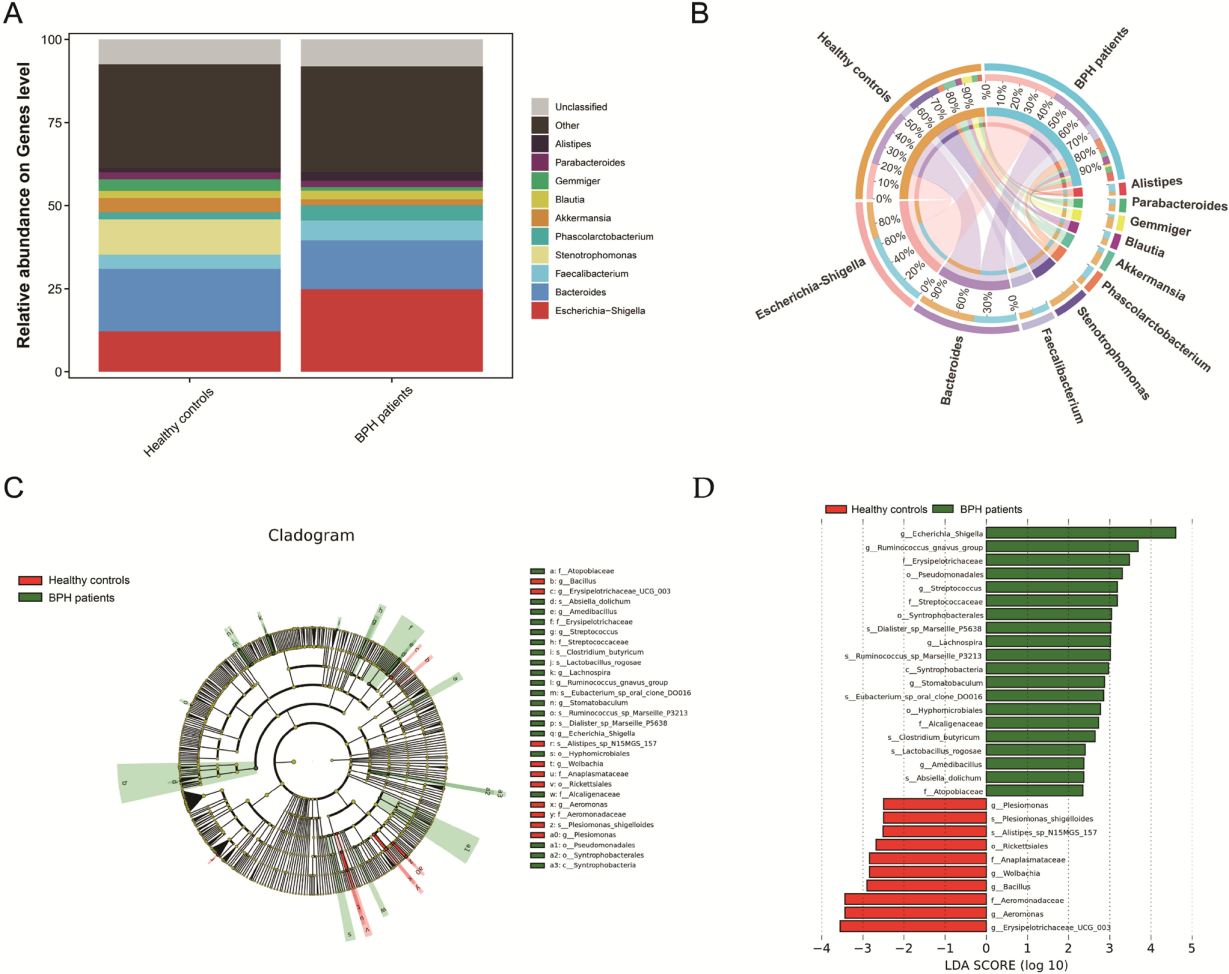
Microbiome profile variations between BPH patients and healthy controls through 16S rRNA sequencing. (A) Bar plot showing the relative genus‐level composition in BPH patients and healthy controls; (B) Circos plot depicting associations between the abundance of dominant genera and the BPH group; (C) LEfSe‐generated cladogram illustrating differences in bacterial taxa between groups; (D) LDA scores (≥ 2) highlighting taxa with different abundance levels between BPH patients and healthy controls. BPH, benign prostatic hyperplasia; LDA, linear discriminant analysis; LEfSe, LDA effect size.

After confirming the causal relationship through MR analysis, we further validated our findings using data from 16S rRNA sequencing of fecal samples obtained from both BPH patients and healthy controls. We examined the compositional differences in the GM at the genus levels between the two groups. Notably, the relative abundances of *Escherichia*–*Shigella* were significantly higher in the BPH patients compared to healthy controls (Figure 3A). The circos graph demonstrated the associations between the abundance of each dominant genus and BPH patients groups (Figure 3B). LDA combined with LEfSe identified the ASVs driving the compositional differences in gut microbiota. The LDA value distribution histogram (*p* < 0.05, LDA score ≥ 2) revealed 30 differentially abundant taxa across taxonomic levels, including 20 enriched in BPH patients and 10 in healthy controls. The genus *Escherichia*–*Shigella* was the dominant taxon in the BPH patients group and had the largest LDA score (Figure 3C,D). This consistency between the MR analysis and sequencing data further strengthens the hypothesis that *Escherichia*–*Shigella* may exhibit a detrimental role in the development of BPH.

### 3.5 Demographic and Clinical Characteristics of 58 BPH Patients

A clinical retrospective analysis was carried out to examine the association between the relative abundance of intestinal *Escherichia*–*Shigella* and LUTS severity in BPH patients. The demographics and clinical data of the 58 BPH patients were shown in Table 2. Based on IPSS scores, the samples were categorized into the mild‐to‐moderate group (25 cases) and the severe group (33 cases). We observed statistically significant disparities between these two groups in PV, NLR, and the median relative abundance of the *Escherichia*–*Shigella* genus (*p* < 0.05).

**TABLE 2:** Demographic data and general clinical characteristics of 58 BPH patients.

Variables	Mild‐to‐moderate group (*n* = 25)	Severe group (*n* = 33)	Total (*n* = 58)	*p*
Age, (year)	69.44 ± 7.12	68.73 ± 7.21	69.03 ± 7.12	0.709^a^
Hypertension, *n* (%)				0.890^b^
No	17 (68.00%)	23 (69.70%)	40 (68.97%)	
Yes	8 (32.00%)	10 (30.30%)	18 (31.03%)	
Diabetes, *n* (%)				0.313^b^
No	19 (76.00%)	21 (63.64%)	40 (68.97%)	
Yes	6 (24.00%)	12 (36.36%)	18 (31.03%)	
Prostate volume, (mL)	48.87 (39.24–57.85)	52.35 (46.19–62.53)	53.11 (44.68–59.92)	0.044^c^
TPSA, (ng/mL)	2.78 (2.54–4.04)	3.70 (2.62–5.82)	3.10 (2.55–4.89)	0.086^c^
BMI, (kg/m^2^)	22.93 ± 2.47	23.28 ± 2.33	23.13 ± 2.38	0.586^a^
CRP, (mg/L)	29.54 (8.32–51.05)	28.96 (18.54–50.50)	29.25 (13.86–50.91)	0.163^c^
Post‐voided residual urine, (mL)	44.66 (34.64–62.02)	45.5 (23.27–65.72)	45.29 (26.90–64.80)	0.554^c^
Neutrophil count, (10^9^/L)	7.09 ± 3.37	8.20 ± 3.26	7.72 ± 3.33	0.213^a^
Lymphocyte count, (10^9^/L)	1.46 ± 0.38	1.31 ± 0.45	1.37 ± 0.43	0.174^a^
Monocyte count, (10^9^/L)	0.45 (0.33–0.54)	0.45 (0.35–0.53)	0.45 (0.33–0.54)	0.808^c^
Platelet count, (10^9^/L)	190.08 ± 50.42	192.21 ± 47.10	191.29 ± 48.14	0.869^a^
NLR	4.40 (3.08–7.29)	6.91 (4.13–8.89)	5.85 (3.86–8.65)	0.032^c^
LMR	3.89 ± 1.88	3.25 ± 1.45	3.52 ± 1.66	0.147^a^
PLR	119.66 (104.71–160.67)	139.47 (118.18–195.50)	133.81 (106.47–186.85)	0.177^c^
SIRI	2.03 (1.12–2.77)	2.94 (1.55–4.00)	2.34 (1.46–3.71)	0.087^c^
*Escherichia*–*Shigella* abundance, (%)	1.82 (0.81–3.12)	6.24 (2.55–10.18)	3.21 (1.51–9.35)	< 0.001^c^

Abbreviations: BMI, body mass index; BPH, benign prostatic hyperplasia; CRP, C‐reactive protein; LMR, lymphocyte‐to‐monocyte ratio; NLR, neutrophil‐to‐lymphocyte ratio; PLR, platelet‐to‐lymphocyte ratio; SIRI, system inflammation response index; TPSA, total prostate specific antigen.

^a^ *p* values were calculated with Student's *t*‐test.

^b^ *p* values were calculated with the chi‐squared test or Fisher's exact test.

^c^ *p* values were calculated with the Mann–Whitney *U* test.

### 3.6 Univariate and Multivariate Logistic Regression Analysis of LUTS Severity in BPH Patients

Subsequently, we conducted univariate and multivariate logistic regression analyses (Table 3) to identify clinical factors associated with LUTS severity in BPH patients. Univariate analysis revealed that PV (OR = 1.06, 95% CI: 1.01–1.12, *p* = 0.026), NLR (OR = 1.22, 95% CI: 1.01–1.47, *p* = 0.039), and *Escherichia*–*Shigella* relative abundance (OR = 1.11, 95% CI: 1.01–1.23, *p* = 0.046) were significantly associated with LUTS severity. Multivariate analysis demonstrated that only *Escherichia*–*Shigella* relative abundance (OR = 1.10, 95% CI: 1.01–1.21, *p* = 0.046) remained significant independent predictors of the severity of LUTS. These findings indicate that the relative abundance of *Escherichia*–*Shigella* independently contributes to the severity of LUTS in BPH patients.

**TABLE 3:** Univariable and multivariable analysis for the severity of LUTS in BPH patients.

Characteristic	Univariable			Multivariable		
	OR	95% CI	*p*	OR	95% CI	*p*
Age	0.99	0.92–1.06	0.703			
Hypertension						
No	Ref	Ref				
Yes	0.92	0.30–2.84	0.890			
Diabetes						
No	Ref	Ref				
Yes	1.81	0.57–5.77	0.316			
Prostate volume	1.06	1.01–1.12	0.026	1.05	0.99–1.12	0.097
TPSA	1.34	0.95–1.88	0.095			
BMI	1.06	0.85–1.33	0.579			
CRP	1.01	0.99–1.03	0.311			
Post‐voided residual urine	0.99	0.97–1.01	0.567			
Neutrophil count	1.11	0.94–1.32	0.213			
Lymphocyte count	0.41	0.11–1.48	0.174			
Monocyte count	0.93	0.04–23.77	0.967			
Platelet count	1.00	0.99–1.01	0.866			
NLR	1.22	1.01–1.47	0.039	1.15	0.94–1.42	0.184
LMR	0.79	0.57–1.09	0.149			
PLR	1.01	1.00–1.02	0.147			
SIRI	1.23	0.92–1.65	0.164			
*Escherichia*–*Shigella* abundance	1.11	1.01–1.23	0.046	1.10	1.01–1.21	0.046

Abbreviations: BMI, body mass index; BPH, benign prostatic hyperplasia; CI, confidence interval; CRP, C‐reactive protein; LMR, lymphocyte‐to‐monocyte ratio; LUTS, lower urinary tract symptoms; NLR, neutrophil‐to‐lymphocyte ratio; OR, odds ratio; PLR, platelet‐to‐lymphocyte ratio; SIRI, system inflammation response index; TPSA, total prostate specific antigen.

### 3.7 Predictive Value of *Escherichia*–*Shigella* Relative Abundance for the Severity of LUTS in BPH Patients

**FIGURE 4:**
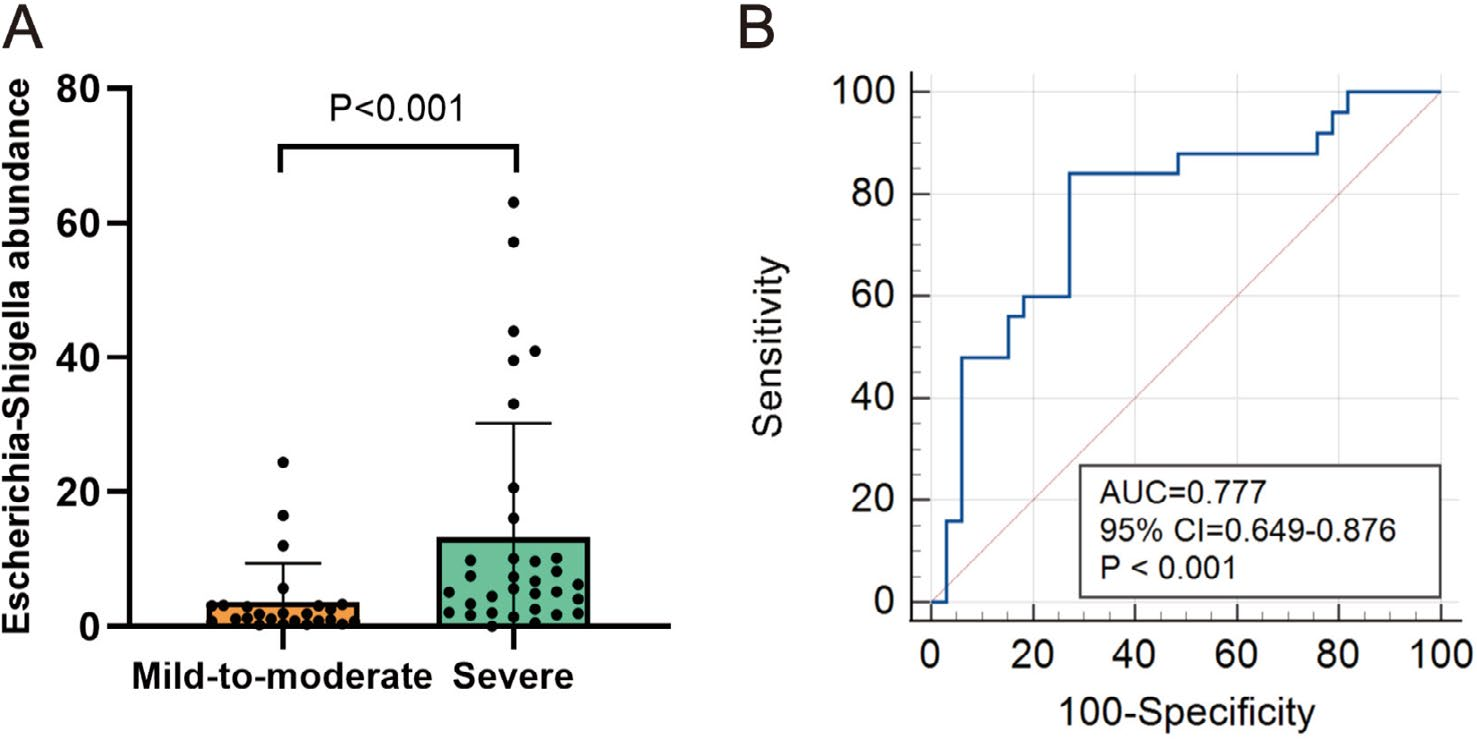
The value of *Escherichia*–*Shigella* relative abundance for predicting LUTS severity in BPH patients. (A) Comparison of the relative abundance of *Escherichia*–*Shigella* between the mild‐to‐moderate group and severe group of LUTS in BPH patients. (B) The ability of *Escherichia*–*Shigella* relative abundance to predict the severity of LUTS in BPH patients. AUC, area under the curve; BPH, benign prostatic hyperplasia; CI, confidence interval; LUTS, the severity of lower urinary tract symptoms.

As shown in Figure 4A, the relative abundance of *Escherichia*–*Shigella* in BPH patients with severe LUTS [6.24 (2.55–10.18)] was notably greater compared to those with mild or moderate LUTS [1.82 (0.81–3.12), *p* < 0.001]. ROC curve analysis (Figure 4B) showed that the relative abundance of *Escherichia*–*Shigella* (AUC = 0.777, 95% CI: 0.649–0.876, *p* < 0.001) may serve as a predictor of LUTS severity in BPH patients. The optimal cutoff values for sensitivity and specificity were 84.00% and 72.73%, respectively. These findings suggest that *Escherichia*–*Shigella* relative abundance holds potential as a predictive marker for the severity of LUTS in BPH patients.

## 4 Discussion

In recent years, considerable research has focused on the human gut microbiota (GM), which evolves in parallel with the host, with the microbiota acquired at birth developing as the host matures (Adak and Khan 2019). It dynamically colonizes the intestinal mucosal layer, and its total number is nearly ten times greater than human cells, with its genome count roughly 150 times that of human genes (Luis and Hansson 2023). The host and GM engage in a bidirectional relationship, with its diversity and abundance impacting the health of the host (Pepke et al. 2024). Emerging evidence has highlighted the potential mechanistic interplay between GM and the pathogenesis of BPH (Pepke et al. 2024; Yue et al. 2024). Previous studies have demonstrated that chronic inflammation plays a key role in the development of BPH (Y. Y. Wang et al. 2024). Since GM is vital for the regulation of systemic inflammation, it may influence the prostate microenvironment through immune‐mediated mechanisms (Liss et al. 2018). Wu et al. (Wu et al. 2025) employed 16S rRNA sequencing to investigate the relationship between Akkermansia levels and LUTS severity in fecal samples from BPH patients and healthy individuals, finding a negative correlation. Li et al. (2022) identified an increase in GM β‐diversity in the gut samples from rats with BPH through high‐throughput sequencing. An et al. (2023) reported that BPH‐ and finasteride‐induced alterations in gut microbiota were intimately associated with the regulation of prostate morphology, hormonal balance, and the programmed death of prostate cells. Therefore, the evolving relationship between the gut microbiota and host health, particularly its role in regulating systemic inflammation, highlights its potential impact on the prevention and treatment of BPH.

As far as we are aware, this is the pioneering study combining two‐sample MR, 16S rRNA sequencing, and clinical retrospective analysis to explore the potential causal relationship between gut microbiota and BPH. MR analyses in our study consistently confirmed that *Phascolarctobacterium*, *Faecalibacterium*, and *Escherichia*–*Shigella* were positively associated with the risk of BPH, while *Lactobacillus* and *Burkholderia* exhibited negative correlations with the risk of BPH. Subsequently, we validated the differences in GM composition between healthy controls and BPH patients through 16S rRNA sequencing of fecal samples. A retrospective clinical analysis was then performed to examine the association between intestinal *Escherichia*–*Shigella* abundance and LUTS severity in BPH. Our findings indicated a positive causal connection between *Escherichia*–*Shigella* abundance and BPH risk, highlighting its potential as an early indicator for BPH and a predictor of LUTS severity.

Based on the findings of this study and existing literature, GM may influence the onset and progression of BPH through various mechanisms. *Lactobacillus*, a key lactic acid producer, is essential for maintaining mucosal integrity and a balanced gut microbiota (Liu et al. 2025), with growing research highlighting its immunomodulatory role in regulating host inflammatory responses (C. Li et al. 2025). An et al. (2023) report that BPH induction reduces *Lactobacillus* abundance in the gut, correlating with reduced prostate apoptosis, while finasteride treatment restores *Lactobacillus* levels, indicating its potential role in regulating prostate health via apoptosis and hormone modulation. This aligns with the findings of our study, which also suggest that *Lactobacillus* has a protective role in BPH, although the exact mechanistic pathways remain to be further explored. *Burkholderiales* is increasingly recognized as a significant modulator of host immunity (Innao et al. 2020). Although direct evidence linking *Burkholderiales* to BPH is limited, its protective role in other inflammation‐related diseases suggests it may exert similar effects in BPH through analogous mechanisms (Chen et al. 2025).

In contrast, *Phascolarctobacterium*, *Faecalibacterium*, and *Escherichia*–*Shigella* may increase the risk of BPH by promoting inflammatory responses or disrupting GM balance. *Phascolarctobacterium* is a Gram‐negative genus within the *Firmicutes* phylum, recognized for its role in propionate production. It may influence systemic inflammation through propionate‐mediated immunoregulatory pathways, potentially affecting cellular proliferation (Huo et al. 2025). *Faecalibacterium*, a major commensal bacterium within the phylum *Firmicutes*, is a predominant producer of the anti‐inflammatory metabolite butyrate in the human gut. Emerging evidence highlights its pivotal role in maintaining gut barrier integrity and immune homeostasis (Geng et al. 2025). Lee et al. (2021) observed that the presence of *Phascolarctobacterium* and *Faecalibacterium* in urine correlates with higher IPSS and more severe storage and voiding symptoms, suggesting their dysbiosis contributes to BPH development and LUTS severity. This observation is consistent with our findings, suggesting that the occurrence and development of BPH may be strongly correlated with the alteration of *Phascolarctobacterium* and *Faecalibacterium*, although the exact causality requires further exploration. *Escherichia*–*Shigella*, a genus within the Gram‐negative *Enterobacteriaceae* family, is a major etiologic agent of bacterial dysentery (Kotloff et al. 2018). Its pathogenicity is primarily attributed to two mechanisms: the induction of intestinal inflammation and the secretion of enterotoxins under inflammatory conditions, both of which can exacerbate dysbiosis and confer a survival advantage to the pathogen (Singh et al. 2015). Previous studies have suggested a potential association between *Escherichia*–*Shigella* and BPH. Xia et al. (2023) conducted a two‐sample MR analysis including 26,358 BPH cases and 110,070 control cases and found that *Escherichia*–*Shigella* may be linked to an elevated risk of BPH. Lee et al. (2021) studied the urinary microbiota of 77 BPH patients and 30 control subjects, finding a notably higher presence of *Escherichia*–*Shigella* in the BPH group than in the controls. While these studies have found higher levels of *Escherichia*–*Shigella* in BPH patients, the underlying causal relationship and mechanistic pathways remain unclear. Our study supports this finding, providing evidence that *Escherichia*–*Shigella* is a key contributor to BPH and a predictor of LUTS severity, confirming its potential role in BPH pathogenesis via systemic inflammation.

As the understanding of the gut microbiome deepens, numerous studies have highlighted the concept of gut‐organ axes and inter‐organ communication between the intestine and other organs (Guadagnoli et al. 2025; Kondapalli et al. 2025; H. Wang et al. 2025). Indeed, the role of GM in prostate diseases has been extensively acknowledged (Song et al. 2025; T. Yang et al. 2025). The “gut‐prostate axis” hypothesis was first introduced in 2005 by Yarnell and Abascal (2005) to describe the close relationship between the prostate and the intestine in the context of prostatitis treatment. Furthermore, Li et al. (2022) constructed a BPH rat model and employed 16S rDNA sequencing and liquid chromatography tandem mass spectrometry to explore the relationship between BPH, gut microbiota, and metabolites, proposing the “gut‐genitourinary axis” hypothesis. Despite the considerable distance between the gut and prostate, recent studies have shown that the abundance of specific bacterial species within the GM is associated with BPH (Ferrari et al. 2024; Takezawa et al. 2021). Enrichment of *Escherichia*–*Shigella*, a Gram‐negative bacterium containing lipopolysaccharide (LPS), could impair the gut barrier, facilitating the entry of LPS into the bloodstream (Shu and Mi 2022). This, in turn, may initiate a chronic inflammatory response by activating immune cells, such as through NF‐κB signaling (J. Li, Li, et al. 2024). Upon activation by LPS, the immune system releases pro‐inflammatory cytokines, such as IL‐17, IL‐23, TNF‐α, and IFN‐γ (Ferrari et al. 2024). These circulating inflammatory mediators could stimulate prostatic stromal and epithelial cell proliferation, inhibit apoptosis, and promote smooth muscle hyperactivity—key pathological features in both BPH development and LUTS progression (De Nunzio et al. 2016). Therefore, our findings suggest that *Escherichia*–*Shigella* is not only a potential biomarker but also a contributory factor in the pathogenesis of BPH through systemic inflammatory pathways.

Building on the insights gained from MR, 16S rRNA sequencing, and a clinical retrospective study, our research suggests that *Escherichia*–*Shigella* may represent a novel therapeutic target for the prevention and management of BPH. Alterations in the abundance and composition of *Escherichia*–*Shigella* species could serve as valuable tools for the early detection of BPH and as potential biomarkers for assessing disease risk. Furthermore, interventions aimed at modulating GM, such as probiotics, dietary modifications, or microbiota transplantation, could significantly reduce the risk of BPH onset and help delay its progression. In addition, targeting the metabolic products of *Escherichia*–*Shigella* may offer a promising new avenue for the treatment of BPH. While direct evidence from clinical trials is still lacking, the causal relationships identified in our study provide a strong theoretical foundation for the future development of microbiota‐based therapeutic strategies for BPH.

While our study employed MR to mitigate biases commonly found in observational studies, it still has several limitations. MR relies on three key assumptions: that instrumental variables are strongly and directly associated with the exposure, are unaffected by confounders, and influence the outcome solely through the exposure. However, in complex biological systems, these assumptions may not always hold true. For instance, certain genetic variants may influence multiple biological pathways beyond the gut microbiota, potentially introducing confounding bias. Additionally, although our 16S rRNA validation supports the MR findings, the relatively small sample size may have limited the statistical power and increased the risk of unstable estimates. Therefore, larger and multicenter studies are warranted to confirm our observations. Furthermore, the gut microbiota GWAS data primarily originate from European populations, while the clinical samples are from Chinese patients, highlighting significant population heterogeneity. The GWAS dataset was chosen for its large sample size and statistical power to detect robust BPH genetic associations. Our clinical validation, using 16S rRNA sequencing and data from a Chinese cohort, aimed to assess the generalizability of MR‐inferred causal relationships. This trans‐ancestry approach tests the stability of findings across populations. Future studies incorporating more diverse populations and employing advanced approaches, such as multivariable MR or proteomic integration, may further refine causal inferences and strengthen the robustness of our conclusions.

## 5 Conclusion

Our MR study sheds light on potential causal links between specific GM and BPH, emphasizing the vital role of GM in prostate health. Additionally, we identified *Escherichia*–*Shigella* as a significant contributor, suggesting its role in predicting the severity of LUTS in BPH patients. These results provide novel insights into BPH development and offer valuable perspectives for developing strategies targeting the gut microbiome, meriting further investigation.

## Author Contributions

Writing – original draft: Ting‐Ting Lin, Bo‐Han Lin, Cheng‐Long Zeng, Zhong‐Hua Zhu. Writing – review and editing: Ye‐Hui Chen, Yi‐Cheng Xu. Methodology: Jun‐Ming Zhu, Yang Chen. Formal analysis: Shao‐Hao Chen, Qing‐Shui Zheng. Data curation: Xue‐Yi Xue, Yong Wei, Ning Xu. Conceptualization: Ting‐Ting Lin, Bo‐Han Lin. Visualization: Cheng‐Long Zeng, Zhong‐Hua Zhu. Project administration: Ye‐Hui Chen, Yi‐Cheng Xu.

## Funding

This study was supported by the Natural Science Foundation of Fujian Provincial (Grant 2024J01515) and the Fujian Provincial Health Technology Project (Grant 2023CXA026).

## Ethics Statement

This study was approved by the Ethics Committee of the First Affiliated Hospital of Fujian Medical University, and all patients provided written informed consent for the clinical samples.

## Conflicts of Interest

The authors declare no conflicts of interest.

## Supporting information


**FIGURE S1:** Leave‐one‐out sensitivity analysis between gut microbiota and BPH. (A) *Phascolarctobacterium*; (B) *Faecalibacterium*; (C) *Escherichia*–*Shigella*; (D) *Lactobacillus*; (E) *Burkholderia*. MR, mendelian randomization.

**Table S1:** STROBE‐MR checklist of recommended items to address in reports of Mendelian randomization studies^1 2^.

**Table S2:** Results for reverse MR analysis.

**Table S3:** Results of horizontal pleiotropy and heterogeneity for BPH.

## Data Availability

The data presented in the study is included in the article, and further inquiries can be directed to the corresponding authors.
