# Supplementary figures and images for: Causal Relationship Between Gut Microbiota and Benign Prostatic Hyperplasia: A Two‐Sample Mendelian Randomization Analyses, 16S rRNA Sequencing and Clinical Retrospective Study
Source: Food Sci Nutr. 2025 Nov 21;13(11):e71261. doi: 10.1002/fsn3.71261 (PMC12636935; doi:10.1002/fsn3.71261)

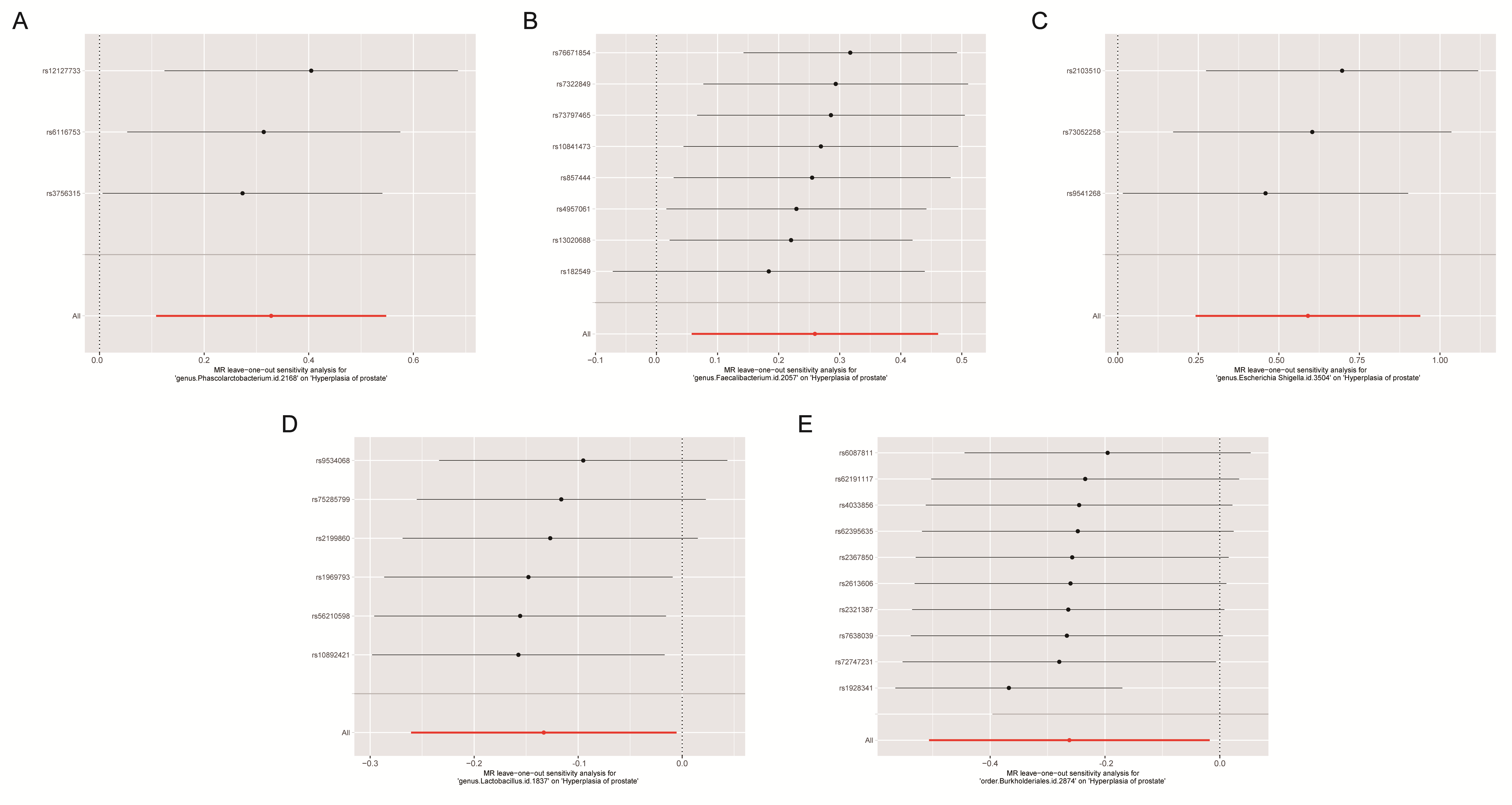

Supplement: Supplementary file 1 — FIGURE S1: Leave‐one‐out sensitivity analysis between gut microbiota and BPH. (A) Phascolarctobacterium; (B) Faecalibacterium; (C) Escherichia–Shigella; (D) Lactobacillus; (E) Burkholderia. MR, mendelian randomization. [file FSN3-13-e71261-s001.png]
