# Supplementary material for: Causal Relationship Between Gut Microbiota and Benign Prostatic Hyperplasia: A Two‐Sample Mendelian Randomization Analyses, 16S rRNA Sequencing and Clinical Retrospective Study
Source: Food Sci Nutr. 2025 Nov 21;13(11):e71261. doi: 10.1002/fsn3.71261 (PMC12636935; doi:10.1002/fsn3.71261)
Supplement: Supplementary file 2 — Table S1: STROBE‐MR checklist of recommended items to address in reports of Mendelian randomization studies1 2. [file FSN3-13-e71261-s004.doc]

**Supplementary Table 1 STROBE-MR checklist of recommended items to address in reports of Mendelian randomization studies^1 2^**

| **Item No.** | **Section** | **Checklist item** | **Page No.** | **Relevant text from manuscript** |
| --- | --- | --- | --- | --- |
| 1 | **TITLE and ABSTRACT** | Indicate Mendelian randomization (MR) as the study’s design in the title and/or the abstract if that is a main purpose of the study | 1-2 | Title: Causal relationship between gut microbiota and benign prostatic hyperplasia: a two-sample Mendelian randomization analyses, 16S rRNA Sequencing and clinical retrospective study  Abstract: We employed Mendelian randomization (MR) to investigate the potential causal links between GM and BPH. |
|  | **INTRODUCTION** |  |  |  |
| 2 | **Background** | Explain the scientific background and rationale for the reported study. What is the exposure? Is a potential causal relationship between exposure and outcome plausible? Justify why MR is a helpful method to address the study question | 5-6 | GM is essential for human health, influencing immune reactions, metabolic functions, development, and various physiological activities. It is also associated with the progression of multiple diseases, including periodontal disease, cancer, diabetes, liver disease, inflammatory bowel disease, and obesity-related disorders(Ditto et al., 2021; Lopetuso et al., 2023). However, various studies have linked differences between healthy and bioimbalanced GM to BPH. Gu et al.(Tsai et al., 2022) reported that high-fat diet-induced GM alterations promoted BPH development. Takezawa et al.(Takezawa et al., 2021) observed significant shifts in GM composition in BPH patients, with Firmicutes and Bacteroidetes markedly enriched. Yang et al.(Yang et al., 2024) suggested that host-mediated modulation of specific GM and engagement of multiple metabolic pathways may underlie the therapeutic effects of Xiaojin pill in BPH. Collectively, these findings indicate that GM alterations may influence the pathogenesis, diagnosis, and early prevention of BPH, while causal relationships remain undetermined.  Although RCTs are regarded as the gold standard for establishing causality, no studies have investigated the relationship between GM and BPH. In Mendelian randomization (MR), genetic variants are used as instrumental variables (IVs) to assess causality between exposures and outcomes. Since alleles are fixed at conception and randomly assorted according to Mendelian principles, MR is less vulnerable to environmental confounding and reverse causation(Ouyang, Liu, Liu, Lan, & Liu, 2024). Consequently, MR is widely applied as an alternative to RCTs in causal inference research. |
| 3 | **Objectives** | State specific objectives clearly, including pre-specified causal hypotheses (if any). State that MR is a method that, under specific assumptions, intends to estimate causal effects | 6 | In our research, we utilized a two-sample MR approach to explore the causal links between specific GM and BPH risk. Subsequently, we employed 16S rRNA gene sequencing to evaluate the relative abundance of GM confirmed by MR analysis in BPH patients. Finally, we explored its relationship with the severity of LUTS, aiming to reveal new aspects of BPH pathogenesis and aid in formulating new preventive and therapeutic strategies. |
|  | **METHODS** |  |  |  |
| 4 | **Study design and data sources** | Present key elements of the study design early in the article. Consider including a table listing sources of data for all phases of the study. For each data source contributing to the analysis, describe the following: |  |  |
|  | a) | Setting: Describe the study design and the underlying population, if possible. Describe the setting, locations, and relevant dates, including periods of recruitment, exposure, follow-up, and data collection, when available. | 6 | Subsequently, fecal samples from 58 BPH patients and 58 healthy male controls, admitted to the First Affiliated Hospital of Fujian Medical University between September 2024 and May 2025, were collected for 16S rRNA sequencing to validate the MR findings. |
|  | b) | Participants: Give the eligibility criteria, and the sources and methods of selection of participants. Report the sample size, and whether any power or sample size calculations were carried out prior to the main analysis | 6-7 | Subsequently, fecal samples from 58 BPH patients and 58 healthy male controls, admitted to the First Affiliated Hospital of Fujian Medical University between September 2024 and May 2025, were collected for 16S rRNA sequencing to validate the MR findings.  The summary statistics for SNPs related to GM were derived from the MiBioGen consortium GWAS database (<https://mibiogen.gcc.rug.nl/>)(Kurilshikov et al., 2021). This multi-ethnic cohort includes 18,340 participants of primarily European descent from 24 cohorts across 11 countries, with 16S rRNA gene sequencing and genotyping data. Locus-based analyses identified GM features, including microbial composition, taxonomic groups, and host genetic variations, while adjusting for sex, age, technical covariates, and genetic principal components. The dataset covered 211 gut microbiota taxa, but 15 unclassified taxa left 196 included taxa. |
|  | c) | Describe measurement, quality control and selection of genetic variants | 7-8 | To assess the causal effect of genetic variants on BPH, we first selected SNPs significantly associated with the exposure (P < 5*10-6) as IVs. |
|  | d) | For each exposure, outcome, and other relevant variables, describe methods of assessment and diagnostic criteria for diseases | 7, 10 | The summary-level GWAS data for BPH were sourced from the FinnGen consortium (ID: finn-b-N14_PROSTHYPERPLA) (ICD code: ICD10: N40 Hyperplasia of prostate) (Kurki et al., 2023).  This validated seven-item questionnaire is designed to assess symptoms such as incomplete emptying, frequency, intermittency, urgency, weak stream, straining, and nocturia. Each item is scored from 0 to 5 based on symptom frequency, resulting in a total score range of 0–35, with higher scores indicating a greater symptom burden. In our study, patients were categorized as mild-to-moderate group (score 0–19) or the severe group (score ≥20). |
|  | e) | Provide details of ethics committee approval and participant informed consent, if relevant | 7 | All participants provided written informed consent for separate studies approved by the ethics committees of the Institutional Review Boards. |
| 5 | **Assumptions** | Explicitly state the three core IV assumptions for the main analysis (relevance, independence and exclusion restriction) as well assumptions for any additional or sensitivity analysis | 6 | MR analysis is based on three essential assumptions: (1) IVs must be strongly associated with the exposure; (2) IVs should be independent of confounders; and (3) IVs should influence the outcome solely through their effect on the exposure(Verduijn, Siegerink, Jager, Zoccali, & Dekker, 2010). |
| 6 | **Statistical methods: main analysis** | Describe statistical methods and statistics used | 8 | Three weighting approaches were applied to examine the association between exposures and outcomes: the inverse-variance weighted (IVW) method, MR-Egger regression, and the weighted median (WM) method. |
|  | a) | Describe how quantitative variables were handled in the analyses (i.e., scale, units, model) | 10-11 | Normally distributed data are presented as mean ± standard deviation (SD), non-normally distributed data as median (interquartile range, IQR), and categorical variables as counts (percentages). |
|  | b) | Describe how genetic variants were handled in the analyses and, if applicable, how their weights were selected | 7-8 | F-statistics were then calculated for each SNP to assess instrument strength, and variants with F ≤10 were excluded to decrease the likelihood of weak-instrument bias. |
|  | c) | Describe the MR estimator (e.g. two-stage least squares, Wald ratio) and related statistics. Detail the included covariates and, in case of two-sample MR, whether the same covariate set was used for adjustment in the two samples | 8 | Three weighting approaches were applied to examine the association between exposures and outcomes: the inverse-variance weighted (IVW) method, MR-Egger regression, and the weighted median (WM) method. |
|  | d) | Explain how missing data were addressed | N/A | N/A |
|  | e) | If applicable, indicate how multiple testing was addressed | 8-9 | To further clarify the criteria for identifying significant findings, we defined a causal relationship as statistically significant when the IVW method produced a P value < 0.05 and the direction of the effect (β) was consistent across at least one complementary approach (MR-Egger regression or WM). The IVW method, as the primary estimator, provides the most efficient and unbiased estimates when all IVs are valid. To enhance the reliability of the results, MR-Egger and WM analyses were conducted as supplementary sensitivity approaches, as they are more tolerant of potential invalid instruments or horizontal pleiotropy. When the direction of β values among different methods was inconsistent, the results were interpreted cautiously. In such cases, the IVW estimate was prioritized due to its higher efficiency under the assumption of no horizontal pleiotropy. However, inconsistent effect directions were viewed as potential indications of bias or pleiotropy, and such associations were not considered robust unless supported by convergent evidence from MR-Egger intercept, MR-PRESSO global tests, and heterogeneity analyses. These combined criteria ensured that only stable and biologically plausible causal signals were retained for interpretation. |
| 7 | **Assessment of assumptions** | Describe any methods or prior knowledge used to assess the assumptions or justify their validity | 10 | To assess the robustness of the results, we performed sensitivity analyses, including tests for heterogeneity, horizontal pleiotropy, and leave-one-out analysis. Heterogeneity for each SNP was evaluated using Cochran’s Q test(Bowden & Holmes, 2019) |
| 8 | **Sensitivity analyses and additional analyses** | Describe any sensitivity analyses or additional analyses performed (e.g. comparison of effect estimates from different approaches, independent replication, bias analytic techniques, validation of instruments, simulations) | 10 | Sensitivity analysis |
| 9 | **Software and pre-registration** |  |  |  |
|  | a) | Name statistical software and package(s), including version and settings used | 10 | All analyses were conducted using the "TwoSampleMR" and "MRPRESSO" packages in R (v4.4.1). |
|  | b) | State whether the study protocol and details were pre-registered (as well as when and where) | N/A | N/A |
|  | **RESULTS** |  |  |  |
| 10 | **Descriptive data** |  |  |  |
|  | a) | Report the numbers of individuals at each stage of included studies and reasons for exclusion. Consider use of a flow diagram |  | Figure 1 |
|  | b) | Report summary statistics for phenotypic exposure(s), outcome(s), and other relevant variables (e.g. means, SDs, proportions) |  | Table 2 |
|  | c) | If the data sources include meta-analyses of previous studies, provide the assessments of heterogeneity across these studies | N/A | N/A |
|  | d) | For two-sample MR:  i.  Provide justification of the similarity of the genetic variant-exposure associations between the exposure and outcome samples  ii.  Provide information on the number of individuals who overlap between the exposure and outcome studies | 7 | The summary-level GWAS data for BPH were sourced from the FinnGen consortium (ID: finn-b-N14_PROSTHYPERPLA) (ICD code: ICD10: N40 Hyperplasia of prostate) (Kurki et al., 2023).  The summary statistics for SNPs related to GM were derived from the MiBioGen consortium GWAS database (<https://mibiogen.gcc.rug.nl/>)(Kurilshikov et al., 2021). |
| 11 | **Main results** |  |  |  |
|  | a) | Report the associations between genetic variant and exposure, and between genetic variant and outcome, preferably on an interpretable scale |  | Table 1 |
|  | b) | Report MR estimates of the relationship between exposure and outcome, and the measures of uncertainty from the MR analysis, on an interpretable scale, such as odds ratio or relative risk per SD difference | 11 | A two-sample MR analysis of 196 GM taxa identified five with a potential causal relationship to BPH risk (Table 1). IVW analysis identified Phascolarctobacterium [odds ratio (OR)=1.286, 95%CI: 1.023-1.618, P=0.031], Faecalibacterium (OR=1.134, 95%CI: 1.008-1.275, P=0.037), and Escherichia-Shigella (OR=1.348, 95%CI: 1.121-1.621, P=0.002) were positively associated with the risk of BPH. |
|  | c) | If relevant, consider translating estimates of relative risk into absolute risk for a meaningful time period | N/A | N/A |
|  | d) | Consider plots to visualize results (e.g. forest plot, scatterplot of associations between genetic variants and outcome versus between genetic variants and exposure) |  | Figure 1-4 and Table 1-3 |
| 12 | **Assessment of assumptions** |  |  |  |
|  | a) | Report the assessment of the validity of the assumptions | 11-12 | Cochran’s Q test was performed on the five GM taxa potentially causally associated with BPH, and neither the IVW nor Egger-intercept methods showed significant heterogeneity (Supplementary Table 3). Egger-intercept results indicated no horizontal pleiotropy between GM and BPH (Supplementary Table 3). Leave-one-out sensitivity analysis revealed no SNPs with a substantial effect on the effect size, indicating the stability of the results (Supplementary Figure 1). Additionally, the MR-PRESSO approach was employed to further evaluate whether there were any outliers or horizontal pleiotropy among the IVs. The results showed that MR-PRESSO did not detect any significant outlier IVs, and none of the models indicated significant horizontal pleiotropy or bias in the estimates. These findings suggest that the selected IVs are appropriate, and the MR estimates are both stable and reliable. The global test results for each microbiota group, as assessed by MR-PRESSO, yielded P-values > 0.05, with the detailed results provided in Supplementary Table 3. |
|  | b) | Report any additional statistics (e.g., assessments of heterogeneity across genetic variants, such as *I^2^*, Q statistic or E-value) | 11-12 | Cochran’s Q test was performed on the five GM taxa potentially causally associated with BPH, and neither the IVW nor Egger-intercept methods showed significant heterogeneity (Supplementary Table 3). Egger-intercept results indicated no horizontal pleiotropy between GM and BPH (Supplementary Table 3). |
| 13 | **Sensitivity analyses and additional analyses** |  |  |  |
|  | a) | Report any sensitivity analyses to assess the robustness of the main results to violations of the assumptions |  | Supplementary Table 3 |
|  | b) | Report results from other sensitivity analyses or additional analyses | 12 | Leave-one-out sensitivity analysis revealed no SNPs with a substantial effect on the effect size, indicating the stability of the results (Supplementary Figure 1). |
|  | c) | Report any assessment of direction of causal relationship (e.g., bidirectional MR) | 11 | The reverse MR analysis yielded no definitive evidence for a causal association between BPH and the five GM taxa (Supplementary Table 2). MR-Egger and WM results were consistent with IVW, supporting the absence of significant causal effects. |
|  | d) | When relevant, report and compare with estimates from non-MR analyses | N/A | N/A |
|  | e) | Consider additional plots to visualize results (e.g., leave-one-out analyses) |  | Supplementary Figure 1 |
|  | **DISCUSSION** |  |  |  |
| 14 | **Key results** | Summarize key results with reference to study objectives | 14 | As far as we are aware, this is the pioneering two-sample MR study examining the causal connection between GM and BPH, leveraging publicly accessible genetic databases combined with 16S rRNA sequencing of clinical patient fecal samples. MR analyses in our study consistently confirmed that Phascolarctobacterium, Faecalibacterium, and Escherichia-Shigella were positively associated with the risk of BPH, while Lactobacillus and Burkholderia exhibited negative correlations with the risk of BPH. Subsequently, we validated the differences in GM composition between healthy controls and BPH patients through 16S rRNA sequencing of fecal samples. A retrospective clinical analysis was then performed to examine the association between intestinal Escherichia-Shigella abundance and LUTS severity in BPH. Our findings indicated a positive causal connection between Escherichia-Shigella abundance and BPH risk, highlighting its potential as an early indicator for BPH and a predictor of LUTS severity. |
| 15 | **Limitations** | Discuss limitations of the study, taking into account the validity of the IV assumptions, other sources of potential bias, and imprecision. Discuss both direction and magnitude of any potential bias and any efforts to address them | 17-18 | While our study employed MR to mitigate biases commonly found in observational studies, it still has several limitations. MR relies on three key assumptions: that instrumental variables are strongly and directly associated with the exposure, are unaffected by confounders, and influence the outcome solely through the exposure. However, in complex biological systems, these assumptions may not always hold true. For instance, certain genetic variants may influence multiple biological pathways beyond the gut microbiota, potentially introducing confounding bias. Additionally, although our 16S rRNA validation supports the MR findings, the relatively small sample size may have limited the statistical power and increased the risk of unstable estimates. Therefore, larger and multicenter studies are warranted to confirm our observations. Furthermore, the gut microbiota GWAS data primarily originate from European populations, while the clinical samples are from Chinese patients, highlighting significant population heterogeneity. The GWAS dataset was chosen for its large sample size and statistical power to detect robust BPH genetic associations. Our clinical validation, using 16S rRNA sequencing and data from a Chinese cohort, aimed to assess the generalizability of MR-inferred causal relationships. This trans-ancestry approach tests the stability of findings across populations. Future studies incorporating more diverse populations and employing advanced approaches, such as multivariable MR or proteomic integration, may further refine causal inferences and strengthen the robustness of our conclusions. |
| 16 | **Interpretation** |  |  |  |
|  | a) | Meaning: Give a cautious overall interpretation of results in the context of their limitations and in comparison with other studies | 17 | Building on the insights gained from MR, 16S rRNA sequencing, and a clinical retrospective study, our research suggests that Escherichia-Shigella may represent a novel therapeutic target for the prevention and management of BPH. Alterations in the abundance and composition of Escherichia-Shigella species could serve as valuable tools for the early detection of BPH and as potential biomarkers for assessing disease risk. Furthermore, interventions aimed at modulating GM, such as probiotics, dietary modifications, or microbiota transplantation, could significantly reduce the risk of BPH onset and help delay its progression. In addition, targeting the metabolic products of Escherichia-Shigella may offer a promising new avenue for the treatment of BPH. While direct evidence from clinical trials is still lacking, the causal relationships identified in our study provide a strong theoretical foundation for the future development of microbiota-based therapeutic strategies for BPH. |
|  | b) | Mechanism: Discuss underlying biological mechanisms that could drive a potential causal relationship between the investigated exposure and the outcome, and whether the gene-environment equivalence assumption is reasonable. Use causal language carefully, clarifying that IV estimates may provide causal effects only under certain assumptions | 14-15 | Escherichia-Shigella, a component of the Gram-negative Enterobacteriaceae family, is the most common causative agent of bacterial dysentery in humans(Kotloff, Riddle, Platts-Mills, Pavlinac, & Zaidi, 2018). It serves two crucial functions in the human body: on the one hand, it induces intestinal inflammation; on the other hand, it secretes enterotoxins in an inflammatory environment, exacerbating intestinal microecological disruption to gain a stronger survival advantage(Singh et al., 2015). Xia et al.(Xia et al., 2023) conducted a two-sample MR analysis including 26,358 BPH cases and 110,070 control cases and found that Escherichia-Shigella may be linked to an elevated risk of BPH. Lee and colleagues(Lee et al., 2021) studied the urinary microbiota of 77 BPH patients and 30 control subjects, finding a notably higher presence of Escherichia-Shigella in the BPH group than in the controls. Although previous studies have found higher levels of Escherichia-Shigella in BPH patients, they did not elucidate its specific relationship and mechanism with BPH. Our study identifies Escherichia-Shigella as an important factor affecting BPH and significantly contributes to forecasting LUTS severity in BPH patients. |
|  | c) | Clinical relevance: Discuss whether the results have clinical or public policy relevance, and to what extent they inform effect sizes of possible interventions | 17 | Building on the insights gained from MR, 16S rRNA sequencing, and a clinical retrospective study, our research suggests that Escherichia-Shigella may represent a novel therapeutic target for the prevention and management of BPH. |
| 17 | **Generalizability** | Discuss the generalizability of the study results (a) to other populations, (b) across other exposure periods/timings, and (c) across other levels of exposure | 17 | Our clinical validation, using 16S rRNA sequencing and data from a Chinese cohort, aimed to assess the generalizability of MR-inferred causal relationships. This trans-ancestry approach tests the stability of findings across populations. |
|  | **OTHER INFORMATION** |  |  |  |
| 18 | **Funding** | Describe sources of funding and the role of funders in the present study and, if applicable, sources of funding for the databases and original study or studies on which the present study is based | 25 | This study was supported by the Natural Science Foundation of Fujian Provincial (Grant number: 2024J01515) and the Fujian Provincial Health Technology Project (Grant number: 2023CXA026). |
| 19 | **Data and data sharing** | Provide the data used to perform all analyses or report where and how the data can be accessed, and reference these sources in the article. Provide the statistical code needed to reproduce the results in the article, or report whether the code is publicly accessible and if so, where | 25 | The data presented in the study are included in the article, and further inquiries can be directed to the corresponding authors. |
| 20 | **Conflicts of Interest** | All authors should declare all potential conflicts of interest | 25 | All authors declare no conflict of interests. |

This checklist is copyrighted by the Equator Network under the Creative Commons Attribution 3.0 Unported (CC BY 3.0) license.

1. Skrivankova VW, Richmond RC, Woolf BAR, Yarmolinsky J, Davies NM, Swanson SA, et al. Strengthening the Reporting of Observational Studies in Epidemiology using Mendelian Randomization (STROBE-MR) Statement. JAMA. 2021;under review.

2. Skrivankova VW, Richmond RC, Woolf BAR, Davies NM, Swanson SA, VanderWeele TJ, et al. Strengthening the Reporting of Observational Studies in Epidemiology using Mendelian Randomisation (STROBE-MR): Explanation and Elaboration. BMJ. 2021;375:n2233.
