# Supplementary material for: Causal Relationship Between Gut Microbiota and Benign Prostatic Hyperplasia: A Two‐Sample Mendelian Randomization Analyses, 16S rRNA Sequencing and Clinical Retrospective Study
Source: Food Sci Nutr. 2025 Nov 21;13(11):e71261. doi: 10.1002/fsn3.71261 (PMC12636935; doi:10.1002/fsn3.71261)
Supplement: Supplementary file 3 — Table S2: Results for reverse MR analysis. [file FSN3-13-e71261-s003.doc]

**Supplementary Table 2 Results for reverse MR analysis**

| **Gut microbiota** | **Method** | **nSNP** | **OR（95%CI）** | **P value** |
| --- | --- | --- | --- | --- |
| Phascolarctobacterium | IVW | 13 | 0.992（0.934-1.053） | 0.781 |
|  | MR-Egger | 13 | 1.030（0.803-1.321） | 0.822 |
|  | WM | 13 | 0.978（0.913-1.046） | 0.513 |
| Faecalibacterium | IVW | 5 | 1.024（0.975-1.075） | 0.337 |
|  | MR-Egger | 5 | 1.124（0.925-1.362） | 0.266 |
|  | WM | 5 | 1.041（0.978-1.109） | 0.229 |
| Escherichia-Shigella | IVW | 4 | 1.043（0.926-1.171） | 0.482 |
|  | MR-Egger | 4 | 1.222（1.015-1.475） | 0.067 |
|  | WM | 4 | 0.972（0.855-1.105） | 0.665 |
| Lactobacillus | IVW | 7 | 0.949（0.875-1.030） | 0.285 |
|  | MR-Egger | 7 | 1.047（0.770-1.423） | 0.772 |
|  | WM | 7 | 0.941（0.876-1.030） | 0.205 |
| Burkholderiales | IVW | 13 | 1.040（0.975-1.102） | 0.216 |
|  | MR-Egger | 13 | 1.124（0.924-1.368） | 0.262 |
|  | WM | 13 | 1.025（0.975-1.074） | 0.254 |

MR, mendelian randomization; SNP, single nucleotide polymorphism; OR, odds ratio; CI, Confidence Interval; IVW, inverse-variance weighted; WM, weighted median
