# Supplementary material for: Causal Relationship Between Gut Microbiota and Benign Prostatic Hyperplasia: A Two‐Sample Mendelian Randomization Analyses, 16S rRNA Sequencing and Clinical Retrospective Study
Source: Food Sci Nutr. 2025 Nov 21;13(11):e71261. doi: 10.1002/fsn3.71261 (PMC12636935; doi:10.1002/fsn3.71261)
Supplement: Supplementary file 4 — Table S3: Results of horizontal pleiotropy and heterogeneity for BPH. [file FSN3-13-e71261-s002.doc]

**Supplementary Table 3 Results of horizontal pleiotropy and heterogeneity for** BPH

| **Gut microbiota** | **Horizontal pleiotropy** | | | **MR-PRESSO**  **Outlier-corrected** | **Heterogeneity** | | | |
| --- | --- | --- | --- | --- | --- | --- | --- | --- |
|  | **Egger-intercept** | **p-value** | **MR-PRESSO** |  | **MR-Egger’ Q** | **MR-Egger’ P** | **IVW’ Q** | **IVW’ P** |
| Phascolarctobacterium | -0.011 | 0.592 | 0.536 | NA | 2.596 | 0.412 | 2.885 | 0.456 |
| Faecalibacterium | -0.019 | 0.620 | 0.633 | NA | 3.065 | 0.339 | 3.691 | 0.442 |
| Escherichia-Shigella | 0.055 | 0.712 | 0.725 | NA | 1.992 | 0.595 | 1.875 | 0.617 |
| Lactobacillus | 0.014 | 0.577 | 0.598 | NA | 4.572 | 0.354 | 5.022 | 0.266 |
| Burkholderiales | 0.019 | 0.675 | 0.688 | NA | 6.364 | 0.669 | 6.903 | 0.702 |

BPH, benign prostatic hyperplasia; MR, mendelian randomization;IVW, inverse-variance weighted
